# Chalcopyrite Nanoparticles as a Sustainable Thermoelectric Material

**DOI:** 10.3390/nano5041820

**Published:** 2015-10-29

**Authors:** Maninder Singh, Masanobu Miyata, Shunsuke Nishino, Derrick Mott, Mikio Koyano, Shinya Maenosono

**Affiliations:** Japan Advanced Institute of Science and Technology, School of Materials Science, 1-1 Asahidai, Nomi, Ishikawa 923-1292, Japan; E-Mails: s1340014@jaist.ac.jp (M.S.); s1330067@jaist.ac.jp (M.M.); s_nishino@jaist.ac.jp (S.N.); derrickm@jaist.ac.jp (D.M.); koyano@jaist.ac.jp (M.K.)

**Keywords:** thermoelectric, nanomaterial, sustainable, chalcopyrite

## Abstract

In this report, copper iron sulfide nanoparticles with various composition were synthesized by a thermolysis based wet chemical method. These inherently sustainable nanoparticles were then fully characterized in terms of composition, structure, and morphology, as well as for suitability as a thermoelectric material. The merits of the material preparation include a straightforward bulk material formation where particles do not require any specialized treatment, such as spark plasma sintering or thermal heating. The Seebeck coefficient of the materials reveals P-type conductivity with a maximum value of 203 µV/K. The results give insight into how to design and create a new class of sustainable nanoparticle material for thermoelectric applications.

## 1. Introduction

In the field of energy materials, nanotechnology has already found many uses. Nanoparticles and the related nano-fabrication techniques have been used in catalysts for fuel cells [1], in solar panels as the light collecting component [2], for hydrogen production [3] and storage [4], as well as a host of other applications. More recently, nanotechnology has been used in thermoelectric materials, which have the potential to greatly enhance our current energy production efficiency. Thermoelectric materials rely on the Seebeck and Peltier effects to convert an electric current to a heat gradient, or *vice versa*. By utilizing these phenomena, thermoelectric materials can be used to generate electricity from nearly any heat source, for example an automobile engine, in steam turbine electricity generation, or even direct geothermal energy. Until now though, thermoelectric materials have not found widespread use because of their inherently low energy conversion efficiency, described by the dimensionless figure of merit, ZT. In recent years, however, new techniques revolving around nanotechnology have been developed that have opened up the doors to improving the ZT value. Techniques such as nanostructuring allow suppression of the thermal conductivity, which is a useful tool to enhance ZT. These advancements in improving efficiency values have been exciting, yet from the aspect of material sustainability there are still many challenges left to address. The very best thermoelectric materials available today (*i.e.*, Bi_2_Te_3_, BiSbTe_3_, PdTe, *etc.*) contain either rare or toxic elements that limit their practical application [5,6,7,8]. Tellurium is one such element that is present in nearly all of the high efficiency thermoelectric materials because of its beneficial electronic band properties [8,9], but the element is extremely rare on Earth, making these materials increasingly more expensive. With this in mind, new sustainable thermoelectric materials must be sought out that do not rely on rare or toxic elements. To accomplish this, the nanotechnology techniques that have been pioneered in enhancing the thermoelectric efficiency of the traditional materials should now be applied to sustainable materials systems to elucidate and identify new techniques for optimizing thermoelectric properties through the material characteristics such as particle size, shape, composition, structure or interparticle properties [8]. In this work, we developed a synthetic approach toward nanoparticles composed of copper, iron and sulfur, which is attractive because of the abundant nature of the constituent elements. Apart from this abundant nature, the Cu-Fe-S system is chosen for its structural properties, which can prove to be beneficial for good thermoelectric characteristics [10]. Chalcopyrite has a tetragonal structure with lattice constants *a* = 5.289 Å and *c* = 10.423 Å. This class of compound shows a relatively large carrier mobility [11], which is beneficial for thermoelectric performance. The synthetic approach used allows control of the nanoparticle composition by changing the metallic feeding ratio. Copper sulfide (and its related materials) is a widely studied semiconductor material [12,13,14,15], and the particles can be created in a straightforward thermolysis reaction [16,17]. The resulting particle characteristics are studied using techniques, such as transmission electron microscopy (TEM), X-ray diffraction (XRD) and inductively coupled plasma-optical emission spectroscopy (ICP-OES), then the material is processed into a solid pellet to characterize the Seebeck value of the material. The processing of the sample for Seebeck measurement does not include any ligand exchange or thermal treatment of the nanoparticles, which is highly beneficial since it preserves the true nanoparticle size. This fundamental difference for our materials provides a great contrast to the past studies on the thermoelectric materials composed of chalcopyrite. It is also important to note that the predominant Seebeck value for bulk chalcopyrite is N-type [8], which is in contrast to our measured value showing P-type conductivity. While doping of the chalcopyrite material can be used to control the type of conduction, it is also possible that surface effects or quantum confinement contribute in this system as well [10,18], providing further merits to this sustainable nanoparticle system for thermoelectrics.

## 2. Results and Discussion

### 2.1. Morphology

**Figure 1:**
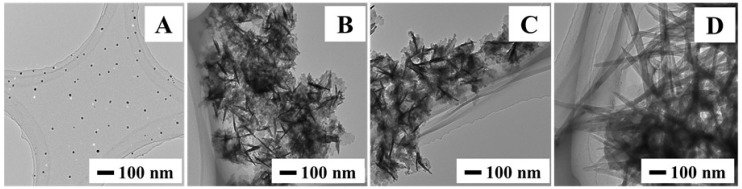
Transmission electron microscopy (TEM) images of nanoparticles synthesized with Cu:Fe metallic feeding ratios of (**A**) 100:0; (**B**) 70:30; (**C**) 50:50; and (**D**) 30:70.

After the synthesis, a dispersion of nanoparticles in methanol was drop-cast on a carbon coated copper micro-grid and was analyzed using TEM. Figure 1 shows the resulting representative TEM image of each set of particles. For sample A (metallic feeding ratio 100% Cu), the particles appear to be spherical in shape with a size of 13.1 ± 2.3 nm. It is likely that these particles are similar to those produced in past studies and are composed of chalcocite with a disk morphology [19]. In this case, the particles are lying on the TEM grid face down. For sample B (metallic feeding ratio of 70% Cu, 30% Fe), however, the particle morphology changes drastically. Some rod-like features can be observed in the image embedded in large clumps. Moving to samples C (metallic feeding ratio of 50% Cu, 50% Fe) and D (metallic feeding ratio of 30% Cu, 70% Fe), the rods become more pronounced and seem to have a central focal point for each cluster. This central point may be the originating nucleation point for each cluster of particles. The clumpy material itself may in fact be sheets of nanoparticle material, which would be consistent with the morphology observed for the parent copper sulfide nanoparticle material [20,21].

### 2.2. Crystalline Properties

**Figure 2:**
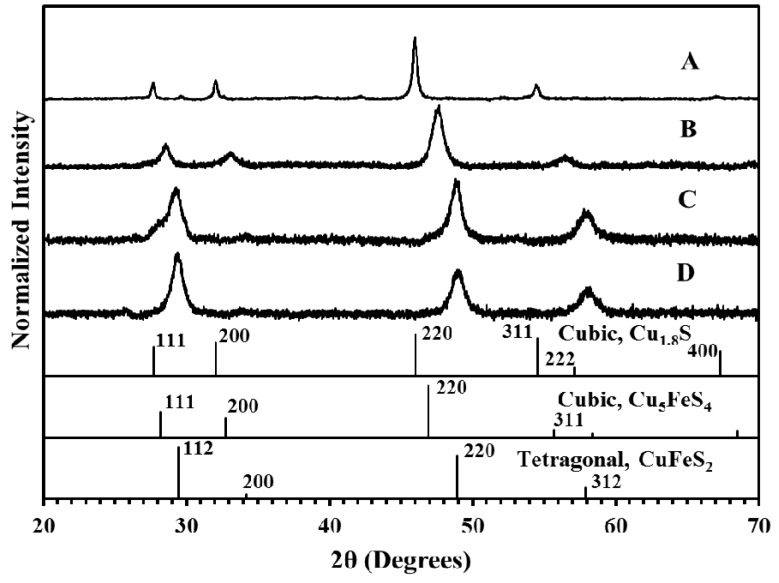
X-ray diffraction (XRD) patterns of nanoparticles synthesized with Cu:Fe metallic feeding ratios of (**A**) 100:0; (**B**) 70:30; (**C**) 50:50; and (**D**) 30:70 [22]. Reference patterns are for PDF card numbers 00-004-0861 (cubic Cu_1.8_S), 01-073-1667 (cubic Cu_5_FeS_4_) and 00-001-0842 (tetragonal CuFeS_2_).

The nanoparticles were next analyzed with XRD to study the crystalline characteristics of the nanoparticles. Figure 2 shows the resulting XRD patterns produced for each sample in the analysis. For sample A, a clear pattern is obtained that matches closely to cubic phase digenite [22]. For sample B, the pattern obtained matches with cubic Cu_5_FeS_4_ [22] phase, which is copper rich, consistent with the metallic feeding ratio used for this sample. For sample C, two phases appear to be present, the primary peaks align closely to the reference locations for tetragonal CuFeS_2_ [22], which is consistent with the metallic feeding ratio used for this sample. In addition, shoulders are observed for both the 112 and 220 peaks, which align with the cubic Cu_5_FeS_4_ phase, indicating the presence of a small amount of this material. Finally, for sample D, the observed peaks match the reference for tetragonal CuFeS_2_ [22], which is not consistent with the fact that the feeding ratio of iron is highest in this case. It is likely that some iron remains unreacted in this sample and is removed with subsequent cleaning after synthesis, or as will be shown later, some iron exists as an amorphous material in the sample which cannot be identified with XRD. As a final qualitative observation, in general the broad peaks observed in samples B, C, and D show that the particles possess a smaller grain size when compared to sample A. This is contrary to the visual inspection of the TEM images, but could be due to the clumpy material in samples B, C and D being composed of very thin sheets, which would lead to the observed peak broadening. In general, we observe a systematic shift from the cubic phase of Cu_1.8_S to cubic Cu_5_FeS_4_, and then to tetragonal CuFeS_2_ as the metallic feeding ratio of iron is increased.

### 2.3. Composition

To gain an understanding of the material composition, ICP-OES was performed for each sample. Nanoparticle (0.5 mg) samples were dissolved in 1 mL of aqua-regia, and then were diluted with distilled water to a volume of 50 mL to achieve a total dilution of about 10 ppm concentration. Calibration curves for copper and iron were created by using ICP standard solutions of each metal at 2, 4, 6, 8 and 10 ppm. The samples were analyzed and the calibration curves were used to calculate the nanoparticle compositions. The material atomic compositions are shown in Table 1. In addition, the nanoparticle compositions were measured using energy dispersive X-ray spectroscopy (EDS) (which is integrated with the TEM technique). The samples, which were cast onto carbon coated copper micro grids for TEM, were used in the EDS analysis. Several areas were selected in each grid which included several nanoparticles. The composition from these areas was averaged. These results are also shown in Table 1. While the composition for most samples matches closely with the observed XRD patterns, sample D appears to contain an excess amount of iron (the XRD pattern matches closely with that of the CuFeS_2_ material while the composition shows a content of about 49% iron). To investigate why the metallic feeding ratio of sample D does not seem consistent with the apparent composition or crystal structure observed with XRD we analyzed the composition of several different areas using EDS. In sample D there are two different types of particle observed, one consisting of the nanorods (Area 1) and separate areas that appear dark and clumpy (Area 2). We independently analyzed the composition of these areas and found that the composition is different for these two types of particles. The nanorods (Area 1) have a composition consistent with the crystal structure observed in XRD (51% Cu and 49% Fe) while the clumpy particles (Area 2) contain much more iron (20% Cu and 80% Fe). Based on these results, we believe that the iron rich areas are amorphous in nature, which is why it is not observed in the XRD pattern. This would also explain where the excess iron in the feeding ratio ends up. Additional data on the EDS composition analysis for sample D is provided in the Supporting Information.

**Table 1:** Inductively coupled plasma–optical emission spectroscopy (ICP-OES) and energy dispersive X-ray spectroscopy (EDS) determined atomic compositions for nanoparticle samples.

Sample	Cu:Fe Input (%)	ICP-OES		EDS (%)		
		Cu (%)	Fe (%)	Cu (%)	Fe (%)	
**B**	70:30	77	23	82 ± 3	18 ± 3	
**C**	50:50	54	46	54 ± 1	46 ± 1	
**D**	30:70	36	64	51 ± 8	49 ± 8	Area 1
				20 ± 5	80 ± 5	Area 2

### 2.4. Seebeck Coefficient Measurement

**Figure 3:**
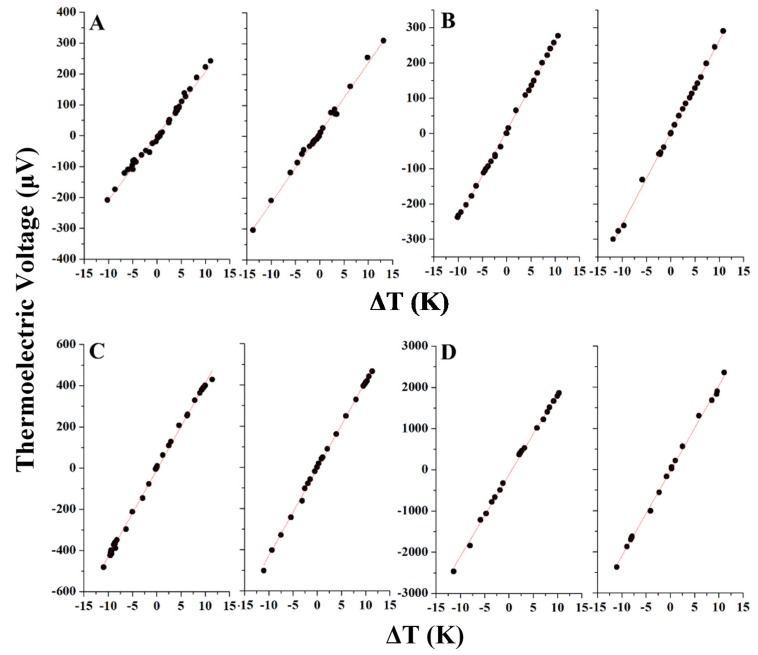
Seebeck measurement plots of sample pellets synthesized with Cu:Fe metallic feeding ratios of (**A**) 100:0; (**B**) 70:30; (**C**) 50:50; and (**D**) 30:70.

In order to measure the Seebeck coefficient of these materials, they must first be processed into a solid pellet. The particles were dried in vacuum and then each sample was pelletized using a hydraulic pellet press. Each sample was pressed with a pressure of 40 MPa for 1 h and pellets with a nominal thickness of ~5 mm and a 10 mm diameter were obtained. The pellet was cut into a square piece using a wire cutter, after this procedure the samples were ready for Seebeck coefficient measurement. The Seebeck measurement was conducted twice for each sample using different pieces of pellet. The pellet piece was fixed onto a glass plate using grease. Two thermocouples (consisting of copper and constantan wires) were fixed onto each side of the pellet using gold paste. The other end of the thermocouple wires were immersed in ice bath to create a reliable reference for temperature (0 °C) and then went on to be attached to a nano-voltmeter to measure the voltage difference between the thermocouples when a heat gradient is created within the pellet. One side of the pellet was heated by contact with a hot soldering gun and the resulting temperature and voltage changes were recorded. The measurement was conducted for two samples. The resulting plots of change in temperature *versus* the thermoelectric voltage reveal the Seebeck coefficient by taking the slope of the fit line of data. Figure 3 shows the representative Seebeck coefficient measurement plots for each sample while Table 2 shows the calculated Seebeck coefficient for each sample taken from two measurements as well as the measured electrical conductivity for each sample. The electrical conductivity provides additional insight into the materials. In general, the electrical conductivity decreases sharply as more iron is incorporated to the particles. The conductivity of the pure copper sulfide is particularly low when compared to reference values, perhaps because these particles have the smallest relative size out of these four samples, providing more interparticle surface area, which will negatively impact the electrical conductivity. These results are consistent with the co-dependent nature of electrical conductivity and Seebeck coefficient. In addition, the power factor was calculated using this data, which is also shown in Table 2. Interestingly the power factor is highest for the copper sulfide (sample A), which is a result of the relatively higher electrical conductivity of this sample. Sample C has the next highest power factor, while sample B and particularly sample D have much lower values. The non-linear nature of the result arises from the fact that Seebeck and electrical conductivity have a co-dependent relationship. A high power factor is obtained for a material with a good balance between Seebeck coefficient and electrical conductivity. In this case, the pure copper sulfide looks most promising, but the thermal conductivity measurements of these materials are also needed to truly judge the overall energy conversion efficiency of these materials. Because of the small amount of material produced in this study, the measurement of thermal conductivity is still a challenge, but is part of the ongoing work. With this in mind, sample C still remains an intriguing possibility to be used as a practical thermoelectric material.

**Table 2:** Seebeck coefficients, electrical conductivity and power factor.

Sample	Seebeck Coefficient (μV/K)	Electrical Conductivity (S/m)	Power Factor (μW/mK^2^)
A	+22 ± 1	9304 ± 539	4.5
B	+26 ± 1	2850 ± 139	1.9
C	+43 ± 1	1519 ± 24	2.8
D	+203 ± 7	2.2 ± 0.4	0.1

### 2.5. Discussion

By using the thermoelectric voltage plots, the Seebeck coefficient was calculated for each sample. We found that sample A (copper sulfide nanoparticles) possesses a Seebeck coefficient of 22 ± 1 µV/K, which is consistent with the reference value for closely related bulk materials such as digenite (Cu_1.8_S) of about 10 µV/K [18]. For sample B the Seebeck coefficient increases to 26 ± 1 µV/K. The Seebeck coefficient then rises again for sample C to 43 ± 1 µV/K and even higher for sample D at 203 ± 7 µV/K. This trend is intriguing given the composition and crystalline properties of these materials. For instance, sample D possesses the highest Seebeck coefficient, yet has a much lower electrical conductivity and corresponding power factor when compared to the other samples. The culprit here is the amorphous material present in sample D, which has very poor electrical conductivity, also causing the power factor to be very low. Since the primary crystalline phases of sample C and D appear to be nearly identical, we can presume that if the amorphous phase of sample D were removed, the electrical conductivity would be greatly enhanced. The Seebeck coefficient may remain the same however, if the crystalline phase is doped with a higher amount of iron in this case, since the Seebeck coefficient measurement is not very sensitive to the overall conductivity quality of the material. Although, we cannot rule out the possibility that the amorphous phase is contributing greatly to the Seebeck coefficient. Experimentally it is a great challenge to separate these two materials to definitively determine this, but a more controlled synthesis of the materials could reveal additional information, which is a part of our ongoing investigations.

All of the samples show P-type conductivity, indicating that the major carrier in these semiconducting materials is holes, which is consistent with copper sulfide materials, but contrasts with the N-type conductivity of bulk chalcopyrite [23]. For example, bulk CuFeS_2_ prepared by a spark plasma sintering technique has a maximum Seebeck coefficient of nearly −600 µV/K at 325 K and is N-type [23], contrary to our own material. However for chalcopyrite nanoparticles with a size of 6.4 nm, the Seebeck coefficient was reported to be over 800 µV/K and P-type [10]. The primary difference between the materials analyzed in this reported study and our own study *versus* bulk chalcopyrite is the nano-sized dimension of the particles. The nano-scale size of the particles used to make the thermoelectric material causes several effects. First, there is a very high degree of particle surface interfaces, and two the small particle size leads to the observation of quantum confinement effects [10,23]. The very high degree of interparticle surface area can affect the Seebeck coefficient by creating a unique local surface composition, for example by being sulfur rich or sulfur deficient [10]. However, the quantum confinement effect may prove to have an even larger impact on the Seebeck coefficient (and other properties) because it will directly alter the electronic properties of the nanoparticles themselves, which is responsible for governing not only Seebeck coefficient, but electrical conductivity as well. While we cannot isolate these phenomena at this time, our results are consistent with past literature reports, and are the objective of part of our ongoing studies on this unique class of sustainable energy material.

## 3. Experimental Section

### 3.1. Chemicals

Tetraethylene glycol 99%, copper (II) nitrate Cu(NO_3_)_2_ 99.999%, iron(II) sulfate FeSO_4_∙7H_2_O ≥99% and thiourea ≥99% were purchased from Sigma Aldrich (Tokyo, Japan). Common solvents including toluene, ethanol and methanol were obtained from Kanto chemical Co. (Kanazawa, Japan). All reagents were used as received without further purification.

### 3.2. Instrumentation

A wide range of instruments were used in the characterization of the nanoparticles studied here. The crystalline properties of the samples were analyzed by powder X-ray diffraction (XRD) with a Rigaku Miniflex instrument and Cu Kɑ radiation (λ = 0.15418 nm). The morphology of the nanoparticle samples were studied with transmission electron microscopy (TEM) using a Hitachi H-7100 instrument operated at 100 kV. TEM samples were prepared by dispersing the dried nanoparticle in methanol and casting a drop of dispersed solution onto a carbon coated Cu grid (from Ted Pella). Sample composition was studied using inductively coupled plasma-optical emission spectroscopy (ICP-OES) using a Shimadzu ICPS-7000 instrument. The Seebeck coefficient for each sample was analyzed at room temperature on a homemade Peltier device [24], the analysis technique is described more fully in the Results Section.

### 3.3. Nanoparticle Synthetic Technique

**Scheme 1:**
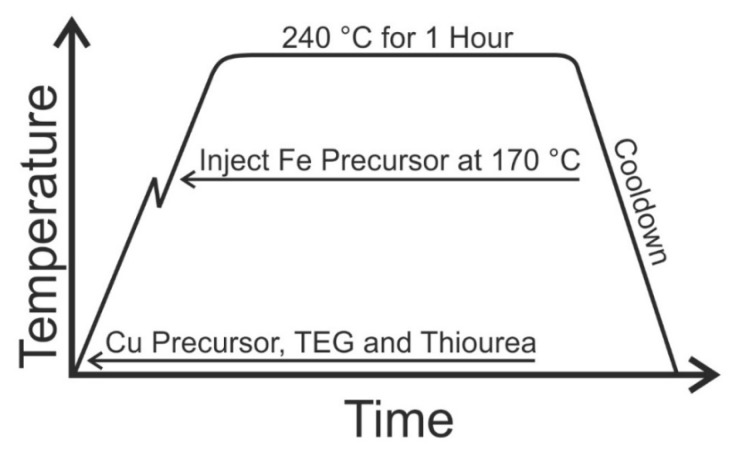
General synthetic technique for Cu-Fe-S nanoparticles.

Cu-Fe-S nanoparticles were synthesized using a modified polyol method as shown in Scheme 1. A total of 10 millimoles of Cu(NO_3_) and Fe(SO_4_)_2_ precursors were used. The molar feeding ratio of copper and iron precursors was varied as shown in Table 3 for a total of 4 samples. Pure iron sulfide nanoparticles were not prepared because the synthetic conditions used here did not lead to the formation of particles. In a typical synthesis, first the copper precursor, 200 mL of tetraethylene glycol as solvent and 12 millimoles of thiourea were added into a three neck round-bottom flask. One of the flask necks was used for monitoring the reaction temperature by thermocouple probe, another neck was fitted with a gas trap and condenser to catch volatile materials during the synthesis and the third neck was used for injecting reactants and for bubbling argon through the reaction solution. The reaction mixture was stirred at 600 rpm using a magnetic stirring bar with argon bubbling and was kept at room temperature for 5 min to remove the air. After that, the flask was heated to 240 °C. When the temperature of the reaction flask reached 170 °C a stock solution containing iron precursor dissolved in 10 mL of methanol was injected into it. The methanol quickly evaporated and was caught in the gas trap. After the reaction temperature reached 240 °C the reaction temperature stabilized and was maintained for one hour. The solution color changed from light grey to black, indicating the formation of nanoparticles. After the reaction, the heating mantle was removed and the reaction mixture was cooled to room temperature. Twenty-five milliliters of methanol were added to the reaction mixture, which was then centrifuged at 5000 rpm for 10 min and the clear supernatant was removed. In this case, we expect the nanoparticles to be coated in thiourea, which would make the particles soluble in polar solvents. After this first washing, the particles were further purified by washing with methanol. The particles were dispersed in 50 mL of methanol with the help of sonication. The mixture was then centrifuged at 5000 rpm for 10 min. The supernatant was removed and the process of washing was repeated three times. The resulting nanoparticles were dried and used for subsequent characterization.

**Table 3:** Input molar ratio of metallic precursors for the Cu-Fe-S nanoparticle system.

Sample	Copper Nitrate (mmol)	Iron Sulfate (mmol)	Cu:Fe (%)
A	10	0	100:0
B	7	3	70:30
C	5	5	50:50
D	3	7	30:70

## 4. Conclusions

In conclusion, we have reported the synthesis and characterization of a sustainable chalcopyrite based nanoparticle system for low temperature thermoelectrics. XRD analysis for these samples shows that the nanoparticles exhibit a phase transition from cubic to tetragonal as the amount of iron is increased in the particles. Compositional analysis shows that the particles contain a composition representative of the feeding ratio, proving that the particle composition can be reliably controlled. The straightforward pellet preparation used in this study allows for the true nanoparticle size to be retained in the thermoelectric materials. Finally, the room temperature Seebeck coefficient was measured for each sample and was found to be P-type for each with a maximum value of 203 ± 7 µV/K for the highest iron content, which is attributed to the high degree of interparticle interfaces and quantum confinement effects arising from the nanoparticle size. The results give insight and provide information into how nanoparticle preparation and processing techniques can be harnessed to create thermoelectric materials with tunable and enhanced characteristics.

## Supplementary Materials

Supplementary materials can be accessed at: http://www.mdpi.com/2079-4991/5/4/1820/s1.
