# Supplementary material for: Chalcopyrite Nanoparticles as a Sustainable Thermoelectric Material
Source: Nanomaterials (Basel). 2015 Oct 29;5(4):1820–30. doi: 10.3390/nano5041820 (PMC5304799; doi:10.3390/nano5041820)
Supplement: Supplementary file 1 [file nanomaterials-05-01820-s001.pdf]

Supplementary Information

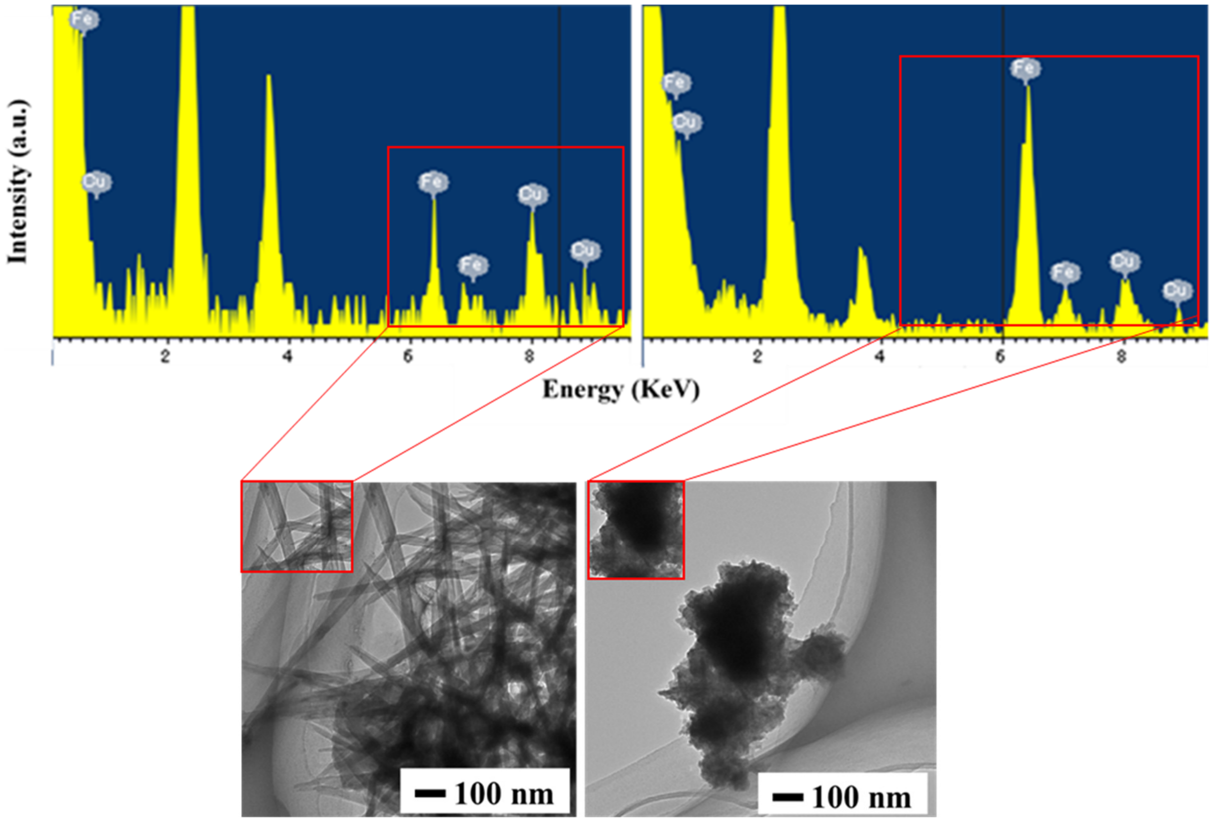

Figure S1. EDS and TEM (above and below) images of nanoparticles synthesized with Cu:Fe metallic feeding ratios of sample D (30:70). Inset TEM image rod like shape (left) and aggregated part (right). The results show the difference in morphology and composition between these two areas in the TEM images. The dark and aggregated material (right side image) is likely iron rich amorphous material.

© 2015 by the authors; licensee MDPI, Basel, Switzerland. This article is an open access article distributed under the terms and conditions of the Creative Commons Attribution license (http://creativecommons.org/licenses/by/4.0/).
